# Reweighting Signal Spectra to Improve Spatial Sensitivity for an Electrostatic Sensor

**DOI:** 10.3390/s19224963

**Published:** 2019-11-14

**Authors:** Jianyong Zhang, Ruixue Cheng, Bing Yan, Mohamed Abdalla

**Affiliations:** 1 School of Computing, Engineering and Digital Technologies, Teesside University, Middlesbrough TS1 3BA, UK; r.cheng@tees.ac.uk (R.C.); mgadwar@gmail.com (M.A.); 2 School of Information and Communication Engineering, North University of China, Taiyuan 030051, China; yanbing122530@126.com

**Keywords:** electrostatic sensor, power spectrum, spatial sensitivity, tail method, velocity profile

## Abstract

The ring-shaped electrostatic sensor is a gas–solid flow measurement system, which has a problem of flow profile dependency. To deal with this problem, a method was introduced in this paper, which was to repeatedly use the successive “tails” of the sensor’s overall output power spectrum to identify elementary frequency components corresponding to the equivalent roping flow streams. From the radial locations of these equivalent flow streams, the decomposed power frequency spectral components were then reweighted accordingly. Through such signal processing, an improved electrostatic sensor spatial sensitivity was achieved without modifying the sensor’s structure. The method of interpolation was presented and discussed, and the effect of velocity profile on the proposed method was evaluated under different velocity profiles.

## 1. Introduction

Ring-shaped electrostatic sensors are used for gas–solid flow measurement, mainly in lean-phase conditions. The key evolutions of this technology in recent decades were well summarized by Gajewski [1]. This type of sensor modeling mainly involves two different aspects. One is the establishment of the relationship between the output and flow rate or concentration considering the effects of various parameters such as velocity and particle size [2,3,4], and the other relates to the dependency of the sensor’s dynamic characteristics on particle distributions [5,6,7,8]. The focus of this paper was on the latter. There have also been developments on modifications of the sensor structure, which have been detailed in other articles [9,10,11]. Several major industrial reviews, including on the state of the art of commercial applications of various gas–solids measurement sensors, have also been published [12,13,14,15,16,17].

In gas–solid two-phase flow, the flow profile describes the distribution pattern of particulates suspended in a gas over a cross-sectional area of pipe [18,19,20]. For a given average concentration, the flow may exhibit various profiles. These flow profiles have generally been classified into homogeneous, stratified, and roping patterns. Unless a sensor isn’t sensitive to flow profile, the measurement results could be different for the same flow rate, depending on the particulate distributions in the pipeline.

It is known that ring-shaped electrostatic sensors have a non-uniform spatial sensitivity. In order to overcome this problem, a two-electrode structure has been proposed [7,21]. This technique requires strictly timed, synchronous signals from two electrodes. Due to the stochastic properties of gas–solid flow signals, this is often impossible, resulting in over- or under-compensation. Another disadvantage of this technique is that the compensation is achieved with a reduced overall sensitivity. Various tomographic sensor arrays [22,23,24] are used to record and reconstruct particle distribution images by reweighting signals according to locations. However, in lean-phase flow, it is difficult to generate useful signals from small electrodes due to low sensitivity and low concentration. If an array of electrostatic electrodes is used for tomographic measurement of flow, its spatial resolution is inherently lower compared with capacitance tomography with the same number of electrodes. In many instances, the objective is simply to measure the solid flow rate or concentration. Considering the cost of manufacturing, maintenance, and the convenience of upgrading technique, the method proposed in this paper has some advantages.

## 2. Modeling

**Figure 1:**
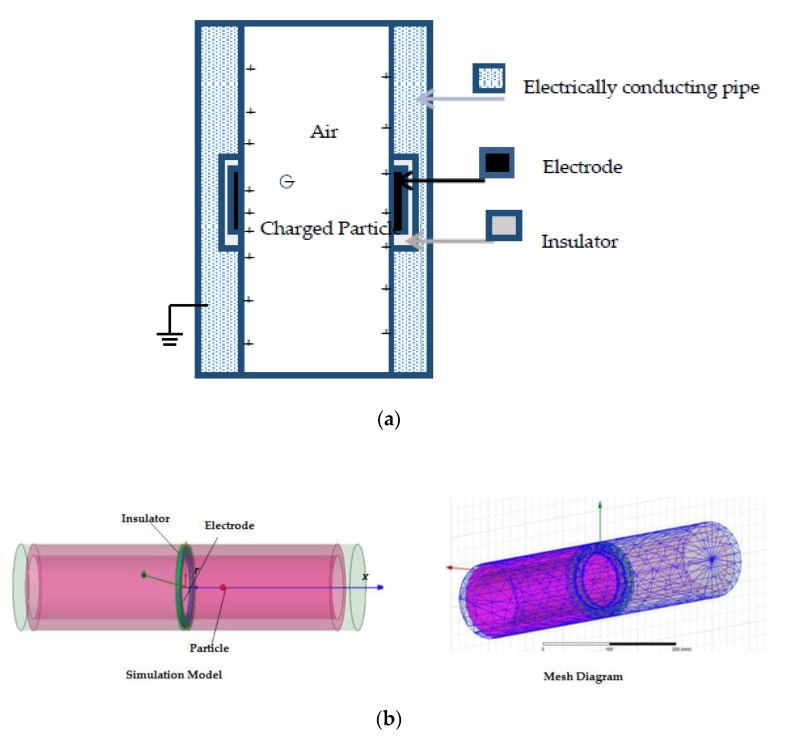
Schematic of ring-shaped electrostatic sensor: (**a**) schematic cross-sectional diagram; (**b**) simulation set-up.

A schematic view of a ring-shaped electrostatic sensor with an inner radius of *R* is depicted in Figure 1a [3]. The ring-shaped metallic electrode is installed flush with and is electrically insulated from the inner surface of the earthed pipe, but is exposed to the flowing air–solid mixture. This arrangement ensures that the electrode is sensitive to the charges carried by particles without restricting flow. It also minimizes the electrode wear compared with an intrusive probe.

During installation, the sensor head is inserted into and electrically connected to the pipeline. Assuming that a single charged particle is transported along pipeline at a radial path *r*, charge induction occurs across the entire inner surface of the pipeline and sensor head. In the state of electrostatic equilibrium, and treating the entire pipeline as an enclosure, the total charge induced on the inner surface of the entire pipeline should equal the charge carried by this particle, based on Gauss’s law. The charge on the entire surface is independent of the location of the particle. However, compared with the entire enclosure, the inner electrode surface used for measurement is only a small fraction of the entire surface; the charge induced on this ring-shaped electrode varies with the location of the particles, and thus the amount of charge induced on the electrode depends upon the axial and radial (*x*, *r*) location of the particle.

The ANSYS Fluent finite element software platform was used for simulation in this paper. The sensor head was assumed to be 300 mm long and 40 mm in diameter with a ring-shaped electrode of 2 mm width. Figure 1b shows the simulation model and mesh diagram.

**Figure 2:**
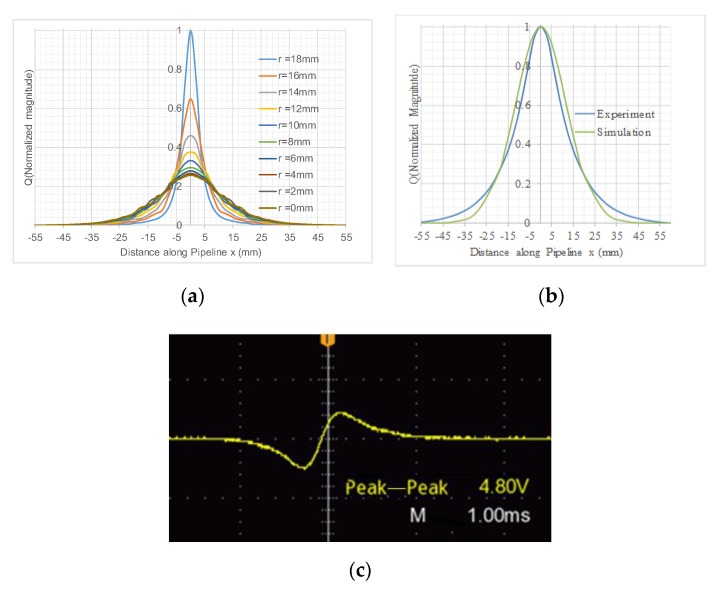
Induced charge seen from different radial paths: (**a**) simulation results; (**b**) comparison to experimental results, *r* = 0 mm; (**c**) experimental results.

Figure 2a depicts the simulated results of induced charge on the electrode due to a single charged particle passing through the sensing zone at radial path *r* = 0 to *r* = 18 mm with the charged particle carrying 1 C (coulomb) charge. As the charge induced on the inner surface of the ring-shaped electrode in the simulation was very low, the results have been normalized for convenience of comparison.

The most commonly used mathematical expressions or models for ring-shaped electrostatic electrodes are Gaussian models [6,7,25], which can be obtained based on the finite element simulation results through curve fitting. The model expressed in Equation (1) was used in this paper, and *A*(*r*) and *k*(*r*) in the equation are functions of *r*.

(1) $$Q_{r}=A\left(r\right)e^{-k\left(r\right)x^{2}}$$

where $Q_{r}$ represents the induced charge on the electrode when a point particle with one unit charge is at location (*x, r*). *x* = 0 is defined as being on the cross-sectional area of the electrode, and *r* = 0 is on the central line of the pipe. Equation (1) is often treated as the expression of spatial sensitivity for this type of sensor; however, in this paper, the spatial sensitivity was defined as the response of the sensor to a stream of particles carrying the same amount of power traveling along the pipeline at different radial paths.

In order to verify the simulation results, experiments were conducted using an electrostatic sensor with the same settings as were used in the simulation: i.e., a 300 mm long sensor body with 40 mm diameter and a 2 mm wide ring-shaped electrode. An air gun was used to shoot a 3 mm diameter plastic bead, and the induced signal was captured with a storage oscilloscope. Based on the mean velocity derived from the two electrodes with a fixed gap and transit time, the distance from the electrode cross-sectional area *x* was determined. The experimental results shown in Figure 2b confirmed that the Gaussian model was able to describe the response of a ring-shaped electrostatic sensor to a charged particle in different locations. The amplitudes of both graphs have been normalized for comparison.

In practice, solids are naturally charged during pneumatic transportation in pipelines. Particle size ranges from a few tens of microns to several millimeters (pulverized to granular). The charge carried by particles depends on their chemical and physical structures, the size of particles, moisture level, and the velocity slip between the two phases.

In the simulation, a point particle with 1 C (coulomb) charge resulted in a charge induction in the range of 10^−7^ to 10^−6^ C (coulomb) on a 2 mm wide, 40 mm diameter electrode when the particle was in the cross-sectional area. The signal received by a digital oscilloscope, shown in Figure 2c, showed the signal when a 3 mm plastic beads passed through the 40 mm diameter meter with the same dimensions and settings as those used for the simulation. The peak to peak voltage of 4.8 V depicted in Figure 2c was proportional to the derivative of the induced charge. The results presented in Figure 2b were produced through integration of the signal in Figure 2c and have been normalized.

From Figure 2a,b, it can be seen that for this 40 mm diameter sensor, the effective sensing zone along the pipeline was within 50 mm of either side of the sensor center’s cross-sectional area. For a typical axial particle velocity of 25 ms^−1^ in the lean-phase pneumatic conveying, the typical transit time for a particle passing the sensing zone was about 4 ms. In such a short distance, the flow profiles over the cross-sectional area of pipe can be regarded as “freeze frames” at a given time in the following analysis. Hence, to express the model in the time domain within the sensing zone, the *x* coordinate in Equation (1) can be replaced with *vt*, where *v* is the particle velocity, *t* is the time, and *t* = 0 is defined as the moment when the particle appears on the electrode’s central cross-sectional area. *Q* is replaced with $Q_{r}\left(t\right)$ in Equation (2).

(2) $$Q_{r}\left(t\right)=A\left(r\right)e^{-k\left(r\right)v^{2}t^{2}}$$

where *A*(*r*) and *k*(*r)* are functions of *r*; *k*(*r*) defines the shape; and *A*(*r*) determines the amplitude of $Q_{r}\left(t\right)$.

As mentioned earlier in this paper, the response of the sensor to a stream of particles passing through the sensing zone at different radial positions, rather than to a single charged particle at location (*x, r*), was used to determine the spatial sensitivity of the sensor, and the stream of the particle at a radial path was regarded as a “roping” flow stream. Hence, the following analysis was done to investigate how the sensor would behave when the same “roping” flow stream traveled through the sensor along different radii.

According to classic control theory, the behavior of a linear system can be evaluated by its unit impulse response. Since the Laplace and Fourier transfer functions of a ring-shaped electrostatic electrode depend on the radial path of the stream of particle flow, as shown in Equation (2), the impulse response of the sensor was analyzed at different paths in this paper. To simplify the analysis in a short sensing zone, it was assumed that at a given time, a stream of particles was evenly spaced along the pipeline and was traveling at velocity *v* in the longitudinal direction along the radial path *r*, with each particle carrying the same amount of charge, *σ_r_*. The total amount of charge contained by the stream, *q_r_*(*t*), can be expressed as

(3) $$q_{r}\left(t\right)=\overset{n_{max}}{\underset{n=-n_{max}}{\sum }}\sigma_{r}\left(t-\frac{n\Delta }{v}\right)$$

where Δ is the gap between two particles, *x* = *n*Δ and *n_max_* = 50 mm/Δ. Here, 50 mm was used as the boundary of the effective sensing zone.

Assuming that at the time *t* = 0, a single particle appears at *x* = 0 with one unit charge,

(4) $$\sigma_{r}{\left(t-\frac{n\Delta }{v}\right)|}_{n=0}=\delta \left(t\right)$$

According to Equation (1), the impulse response *h*(*t*) of the electrode to a single particle is as follows:

(5) $$h\left(t\right)=A\left(r\right)e^{-k\left(r\right)v^{2}t^{2}}$$

The total charge *Q_st_*(*t*) induced on the electrode by a stream of particles is the convolution of *h*(*t*) in Equation (5) and *q_r_*(*t*) in Equation (3).

(6) $$Q_{st}\left(t\right)=h\left(t\right)*q_{r}\left(t\right)=\overset{\infty }{\underset{-\infty }{\int }}h\left(t-\tau \right)q_{r}\left(\tau \right)d\tau$$

Hence, the Fourier transform of *h*(*t*), i.e., *H*(*jω*) in Equation (7), is the transfer function of the ring-shaped electrode. From Equation (5), we have

(7) $$H\left(j\omega \right)=F\left\{h\left(t\right)\right\}=F\left\{A\left(r\right)e^{-k\left(r\right)v^{2}t^{2}}\right\}=\frac{A\left(r\right)\sqrt{\pi }}{2v\sqrt{k\left(r\right)}}e^{-\frac{\omega^{2}}{4k\left(r\right)v^{2}}}$$

where *F*{} represents the Fourier transform operation. $Q_{stF}\left(j\omega \right)$ is the Fourier transform of *Q_st_*(*t*) in Equation (6), giving

(8) $$Q_{stF}\left(j\omega \right)=H\left(j\omega \right)\times q_{rF}\left(j\omega \right)$$

(9) $$q_{rF}\left(j\omega \right)=F\left\{q_{r}\left(t\right)\right\}$$

where the suffix “*F*” denotes that the Fourier transformation $q_{rF}\left(j\omega \right)$ is the Fourier transform of the particle flow stream $q_{r}\left(t\right)$.

## 3. Sensor Output Power Spectrum

In so-called electrostatic dynamic sensors, the conditioning circuit is effectively a differentiator, which performs the first-order derivative to the induced charge on the electrode. As the sensor is the electrode cascaded with the conditioning circuit, therefore, the sensor’s output voltage in the frequency domain, $V_{oF}\left(j\omega \right)$ is as follows:

(10) $$\left|V_{oF}\left(j\omega \right)\right|=\left|H\left(j\omega \right)\times Gj\omega \right|\left|q_{rF}\left(j\omega \right)\right|=\frac{GA\left(r\right)\omega \sqrt{\pi }}{2v\sqrt{k\left(r\right)}}e^{-\frac{\omega^{2}}{4k\left(r\right)v^{2}}}\left|q_{rF}\left(j\omega \right)\right|$$

where *G* is the overall gain of the conditioning circuit and *jω* represents the first order derivative.

In order to link the power of the sensor’s output voltage and the power carried by the charged particles of the flow stream, the power, ${\left|V_{oF}\left(j\omega \right)\right|}^{2}$ was calculated as expressed below, based on Equation (10).

(11) $${\left|V_{oF}\left(j\omega \right)\right|}^{2}=\alpha \left(r\right)\omega^{2}e^{-\beta \left(r\right)\omega^{2}}{\left|q_{rF}\left(j\omega \right)\right|}^{2}$$

and

(12) $$\alpha \left(r\right)={\left|\frac{GA\left(r\right)\sqrt{\pi }}{2v\sqrt{k\left(r\right)}}\right|}^{2},\;\beta \left(r\right)=\frac{1}{2k\left(r\right)v^{2}}$$

The parameters *α*(*r*) and *β*(*r*) were simply introduced for convenience; both are functions of *r*. *α*(*r*) determines the amplitude of the output if one unit of input, i.e., ${\left|q_{rF}\left(j\omega \right)\right|}^{2}=1\;\mathrm{unit}$, is applied at radial path *r*, and *β*(*r*) defines the shape of the output density function.

In this paper, Equation (11) was used to express the spatial sensitivity in terms of the power spectral density function for a given amount of ${\left|q_{rF}\left(j\omega \right)\right|}^{2}$ applied at different radial paths.

In the sensing zone, it was assumed that a stream of evenly spaced particles, each carrying the same amount of charge, traveled along the selected radial paths at the same velocity. The cross-section was divided into 10 rings from *r* = 0 to *r* = 18 mm, with 2 mm width. Based on the simulation results, the values of *α*(*r*) and *β*(*r*) were calculated using Equation (12) at a uniform velocity of 25 ms^−1^, and are provided in Table 1.

**Figure 3:**
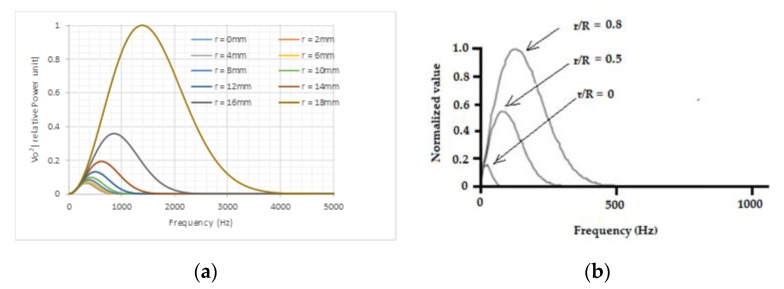
Power density functions when a stream passing different radial paths: (**a**) simulation results; (**b**) experimental results.

The power spectral density functions for this 40 mm diameter sensor with its dynamic conditioning circuit at a uniform velocity of 25 ms^−1^ are shown in Figure 3. As shown in the graphs, it was confirmed that for a particle flow stream with the same power density, i.e., ${\left|q_{rF}\left(j\omega \right)\right|}^{2}=\mathrm{constant}$, the power spectral density of the sensor’s output voltage varied with radial path *r.* This revealed the non-uniform spatial sensitivity of such sensors in the frequency domain.

Figure 3b shows the test results obtained on a 14″ (356 mm) diameter electrostatic sensor [3]. A roping stream with a constant flow rate of about 230 kg/h was provided with a 1 inch jet; the roping stream was parallel to the pipe’s axial central line and moved across the pipe’s cross-sectional area along its diameter. The output voltage (rms) from the output of the sensor conditioning circuit due to the roping flow stream was recorded when the jet moved from the center to a location very close to the pipe wall. The signal was in the range of 20 to 60 mV, which was calculated using the Fourier transform. The power frequency density function obtained from the experiment, shown in Figure 3b, showed a similar trend to the simulation results, although the frequency pass bands of the two were different, which was mainly due to the difference in diameter sizes.

From Figure 3a,b, it is apparent that if the sum of the graphs is used to represent the overall power frequency density of the output, the high-frequency part of the overall curve is always dominated by the output power generated by the outmost layer of flow. It was this observation that formed the base of the output power spectrum decomposition method proposed in this paper, which is explained with examples in the next section.

## 4. Signal Decomposition—Tail Method

A pneumatically conveyed solid flow is commonly regarded as band-limited white noise. Thus, the output signal *V_o_*(*t*) of the conditioning circuit is a stochastic process. Assuming that the sensor’s output signal of the time domain *V_o_*(*t*) is comprised of *V_o1_*(*t*)*, V_o2_*(*t*)*, V_om_*(*t*)*, m* number of uncorrelated stochastic signals due to roping flow streams at all radial paths, and letting the Fourier transform of *V_oi_*(*t*) be *V_oi_*(*jω*), according to Parseval’s theorem below,

(13) $$\overset{+\infty }{\underset{-\infty }{\int }}{\left|V_{o}\left(t\right)\right|}^{2}dt=\frac{1}{2\pi }\overset{+\infty }{\underset{-\infty }{\int }}{\left|V_{oF}\left(j\omega \right)\right|}^{2}d\omega$$

The total power density can be written as the summation as follows:

(14) $$\frac{1}{2\pi }{\left|V_{oF}\left(j\omega \right)\right|}^{2}=\frac{1}{2\pi }\overset{m}{\underset{i=1}{\sum }}{\left|V_{oiF}\left(j\omega \right)\right|}^{2}=\frac{1}{2\pi }{\left|\frac{GA\left(i\Delta r\right)\omega \sqrt{\pi }}{2v\sqrt{k\left(i\Delta r\right)}}e^{-\frac{\omega^{2}}{4k\left(i\Delta r\right)v^{2}}}\right|}^{2}{\left|q_{rF}\left(j\omega \right)\right|}^{2}$$

where *m* = *R*/Δ*r*, and in the calculation Δ*r* = 2 mm, *r* = *i*Δ*r* and *m* = 10.

For a flow stream carrying a certain amount of power, i.e., ${\left|q_{rF}\left(j\omega \right)\right|}^{2}=\mathrm{constant}$, the spectrum density function of the output signal due to this stream, i.e., the spatial sensitivity of the sensor, varies with *r* only, and so does the effective frequency band. Hence, the power, i.e., the area under each curve in Figure 3, needs to be correctly reweighted to achieve the same value for all radial paths. Based on the simulation results, the correction factors were calculated and normalized using the power at *r* = 18 mm as reference, and are provided in Table 2.

As analyzed in the previous sections, if Equation (14) is used to work out the total power directly without using the tail method, errors will be introduced for the measured flow rate or concentration due to the non-uniform spatial sensitivity of the sensor. However, if the component spectra corresponding to the streams at all radial paths are identified from the decomposition of the overall power spectrum, and then the total power for each decomposed component is reweighted according to the correction factors provided in Table 2, the flow measurement will be immune to the flow profile in pipeline. As observed, the tail part of the overall spectrum of the sensor’s voltage output is always dominated by the outmost layer of the flow. If this component is removed from the overall output power spectrum, the tail of the subtotal will be determined by the next outmost layer flow stream. Therefore, this procedure can be repeated until the spectrum generated by the innermost flow stream layer is extracted.

It is very difficult to simultaneously create several flow streams at different radii with different intensities experimentally; hence, this concept was demonstrated and verified using simulation results for the following two examples.

*Example 1*: Assuming that the overall particle flow is comprised of the following streams with the given intensities:

*r* = 0 mm, ${\left|q_{rF}\left(j\omega \right)\right|}^{2}=1$ unit;
*r* = 10 mm, ${\left|q_{rF}\left(j\omega \right)\right|}^{2}=5$ units;
*r* = 14 mm, ${\left|q_{rF}\left(j\omega \right)\right|}^{2}=10$ units;
*r* = 16 mm, ${\left|q_{rF}\left(j\omega \right)\right|}^{2}=6$ units.

**Figure 4:**
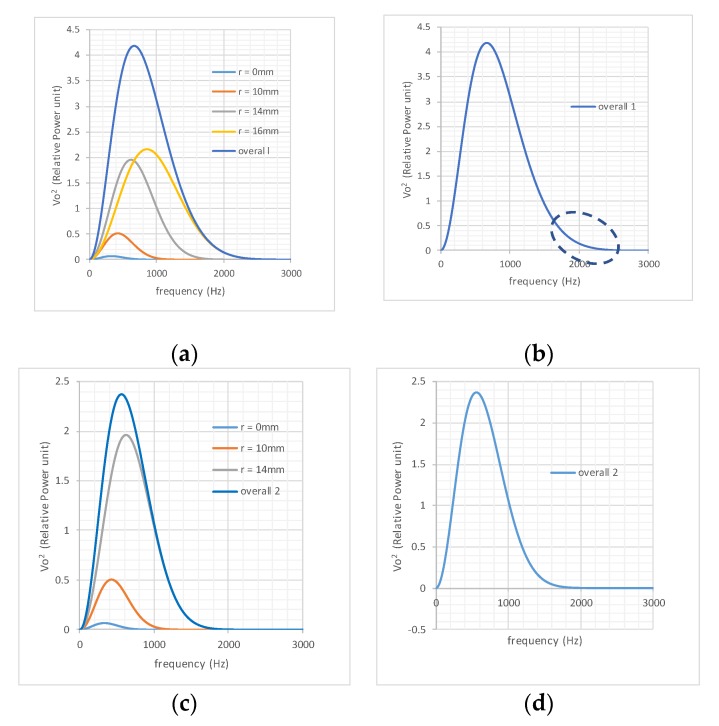
Decomposition procedures for the tail method: (**a**) output power density for each flow stream; (**b**) overall output power density; (**c**) after stripping off the outmost layer; (**d**) new overall output after stripping off the outmost layer.

Figure 4a shows the individual output power spectra of these streams at the above-mentioned radial paths due to the given intensities of the input flow streams. The relative values are used in the graphs, and therefore, the unit for the input is not specified. The sum of the four spectra is shown in Figure 4a. It can be clearly seen that the tail section (high-frequency part) of the overall output is dominated by the response generated by the stream at *r* = 16 mm. Hence, from the tail of “Overall 1”, the power spectral response to the stream at *r* = 16 mm can be identified. Understandably, in a real application, only the overall spectrum would be available, as shown in Figure 4b prior to application of the tail method.

The procedures of the decomposition are as follows.

*A*. Taking 100 points from the tail of Overall 1 in Figure 4b. Assuming that this segment of the graphs satisfies the following function,

(15) $${\left|V_{odF}\left(j\omega \right)\right|}^{2}=\mu \omega^{2}e^{-\beta \omega^{2}}$$

where *μ* is introduced to identify the intensity of the flow stream. ${\left|V_{odF}\left(j\omega \right)\right|}^{2}$ is a decomposed output power spectrum.

*B.* Applying the least-square method using the above 100 points (tail), curve- fitting (non-linear least-square method) was applied to determine the values of factors *μ* and *β* for the first tail, with the calculation accuracy *μ* = 4.911 and *β =* 3.42 × 10^−8^.

*C.* According to the *β* values in Table 1, the radial path for this stream can be identified, which was *r* = 16 mm in this example. According to the *μ* value, the intensity of this flow stream was calculated using the ratio *μ/α* = 4.911/0.818495 = 6, i.e., ${\left|q_{rF}\left(j\omega \right)\right|}^{2}$ was 6 units.

*D.* Stripping off the power spectrum component ${\left|V_{odF}\left(j\omega \right)\right|}^{2}=4.911\omega^{2}e^{-\beta \omega^{2}}$ from Overall 1, a new subtotal output was revealed, indicated by “Overall 2” in Figure 4d. It was clear from Figure 4c that the tail of Overall 2 contained the same information as the power spectrum density corresponding to the stream at *r* = 14 mm.

*E.* Working on the second tail, repeating steps *A* to *D*, *β* = 6.57 × 10^−8^ and *μ* = 8.519 were calculated, which indicated that there a 10 unit flow stream existed at *r* = 14 mm.

The steps from *A* to *E* were repeated until the innermost layer was identified. Table 3 shows the details of the decomposition results produced based on Table 1 and the data from the successive tails shown in Figure 4.

With *μ* and *β* identified was in Table 3*,* integration of the reweighted power spectra using the correction factors in Table 2 should lead to an output which is independent of the particle distribution, i.e., for the same intensity of flow, wherever the radial paths are, the same amount of power will be generated in the output voltage by the sensor, so that a uniform spatial sensitivity is achieved.

The end point of the tail in this example was taken from the 1% of the maximum value of the current overall power density function, and the starting point was found by forwarding the frequency by 100 Hz, with a frequency resolution of 1 Hz. Based on this rule, the start and end points for Tail 1 were 2160 and 2260 Hz, for Tail 2 were 1590 Hz and 1690 Hz, for Tail 3 were 1070 and 1170 Hz, and for Tail 4 were 810 and 910 Hz. In future applications, the starting and ending points will also depend on the frequency resolution and the noise level in the decomposed signal.

*Example 2*: Assume that the flow consists of three streams at *r* = 0 mm, *r* = 2 mm, and *r* = 18 mm, each stream having 1 unit of intensity; ${\left|q_{rF}\left(j\omega \right)\right|}^{2}=1$ unit. There is no flow elsewhere.

**Figure 5:**
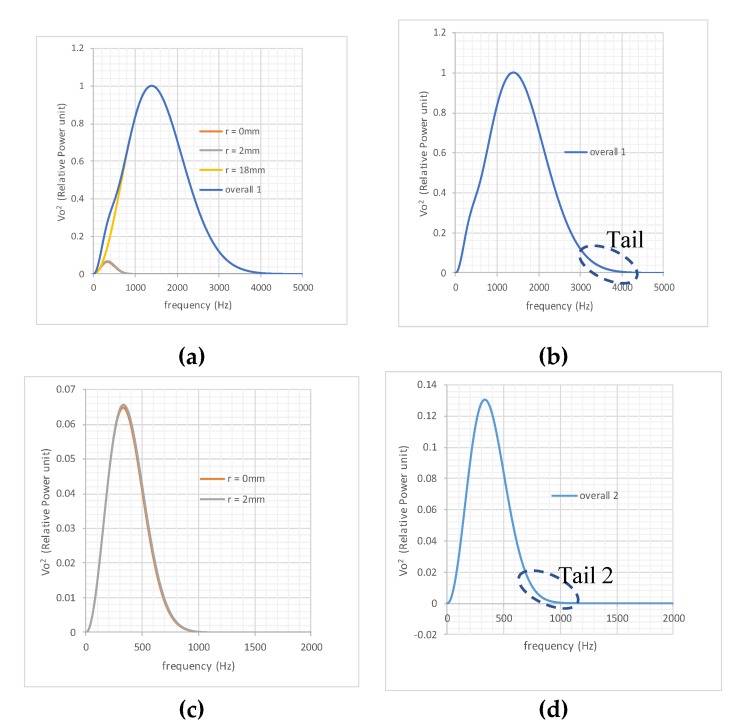
Decomposition with mixed tail data: (**a**) output power density for each flow stream; (**b**) overall output power density; (**c**) output for each flow stream; (**d**) overall output due to adjacent central streams.

Figure 5a shows the three individual spectra and the overall spectrum together, and Figure 5b the overall spectrum only. The tail in Figure 5b was effectively the tail of the outmost layer’s spectrum. Indeed, through the procedures outlined in Example 1, *β* = 1.31 × 10^−8^, and *μ* = 0.8706 were identified, indicating the existence of a flow stream with 1 unit power at *r* = 18 mm.

Figure 5c shows the remaining individual spectra after stripping off the layer at *r* = 18 mm. It can be seen that the remaining two spectra were very close to each other, and almost indistinguishable. Through the procedures, working from the subtotal indicated in Figure 5d, *β* = 2.3047 × 10^−7^ and *μ* = 1.967 were obtained. Referring to the *β* values provided in Table 1, a flow stream between *r* = 0 mm and *r* = 2 mm with an intensity of 1.967 was implied present. The tails of these two spectra lie in a similar frequency band, so the resultant decomposed spectrum did not correspond exactly to either stream, but to a stream between *r =* 0 and *r* = 2 mm with an intensity that almost equaled the summation of the two. This is further discussed in the Interpolation section.

## 5. Interpolation

In the above Example 2, due to the data mixing, there was no corresponding flow stream for a resultant *β* = 2.3047 × 10^−7^ obtained through the tail method. No corresponding radial path *r* can be found in Table 1. In such a case, the interpolation needs to be applied. Furthermore, the simulation or experiments can only be conducted for a limited number of radial paths, and the actual flow streams may appear between the selected paths. Hence, interpolation is always necessary. The interpolation is conducted in the following way: by establishing *r* as a function of *β*, the radial path *r* of the corresponding flow stream can be determined if *β* is obtained from the tail; in turn, with the identified *r,* the power intensity can be determined if the relationship between *α* and *r* has been formed. Table 4 provides the interpolation formulae for the sensor analyzed in this paper.

By applying the interpolation, a flow stream at *r* = 0.9978 mm with 1.97 unit intensity was identified in the above Example 2. At this radial path, *α* was found using the interpolation equation in Table 4, which was 0.998, indicating a flow stream with an intensity of 1.973 units. This represents a 1.34% error, because the actual flow stream power was 2 units.

**Figure 6:**
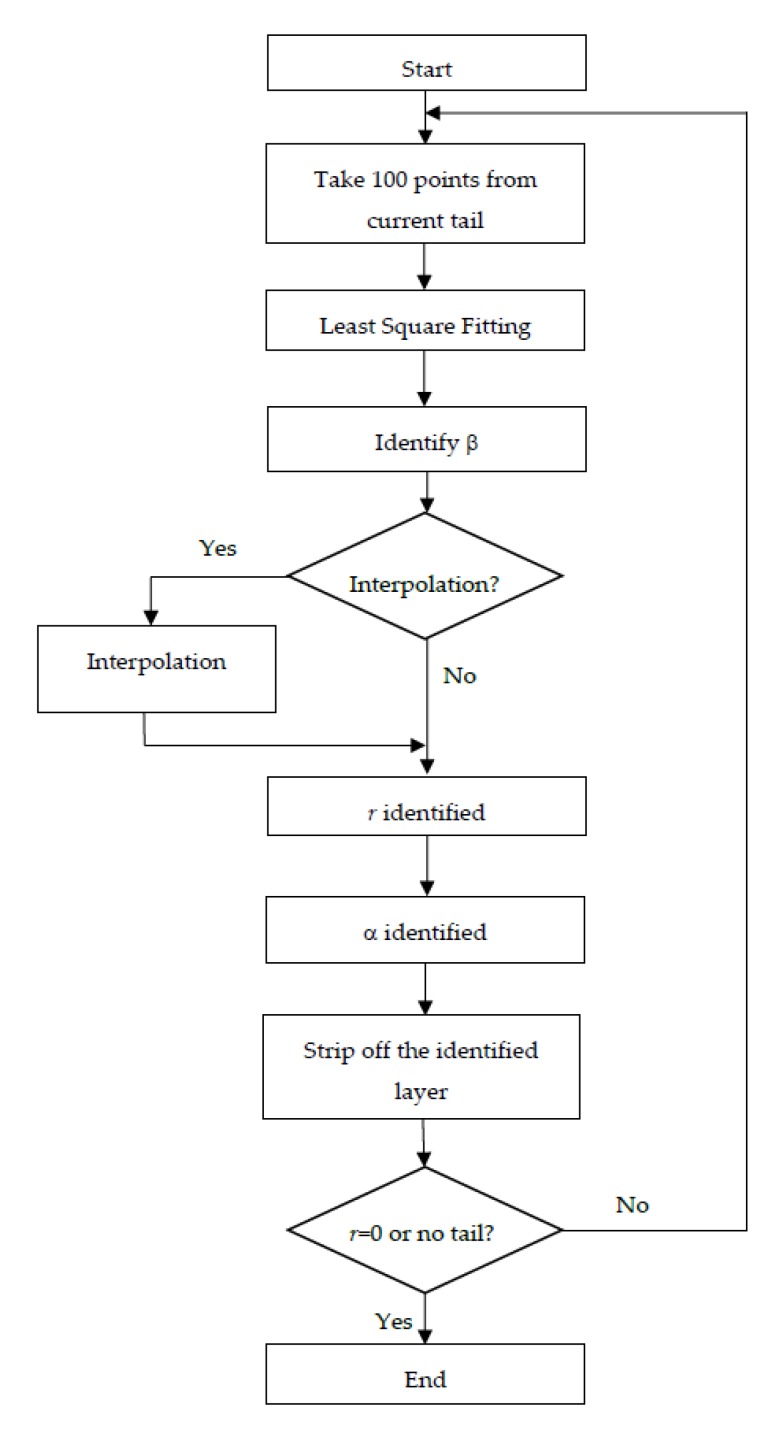
“Tail” method procedure flowchart.

The full procedure of the tail method is presented in the following flowchart (Figure 6).

## 6. Measurement Error Estimation

It is clear that for the same stream of flow through the sensing space at different radial paths, the power spectrum density and total power of the sensor’s output can be very different due to the non-uniform spatial sensitivity of ring-shaped electrostatic sensors. The correction factors provided in Table 2 show that the total power of the output due to a stream passing through *r* = 18 mm is about 64 times that when the same stream travels along *r* = 0 mm, which is the most extreme case.

As the measurement depends on flow profiles, three specific cases were used in the following error estimation: Case 1, even distribution; Case 2, the case presented in Example 1; and Case 3, the flow profile presented in Example 2.

A uniform flow distribution is presented here with a 1 unit flow stream ${\left|q_{rF}\left(j\omega \right)\right|}^{2}=1$, which applies to every radial path from 0 to 18 mm, with 2 mm widths. The total power was obtained by calculating the area under each power density curve. The comparison was conducted between the total power contained by the original signal and the power after the theoretical correction, and the relative error has been given by taking the theoretically corrected total power as the “true” value. However the error caused by data mixing at *r* = 0 mm and *r* = 2 mm was considered, so that the corrected power was not actually error free.

The same method was utilized for Case 2 and case 3. The input flow intensities and the radial paths are specified in Table 5.

It can be seen from Table 5 that in Case 1, where the solids are evenly distributed, the measurement error without compensation could be as high as −85.2%; for Case 2 and Case 3, the errors reached −89.2%, and −65.6%, respectively. Taking into account the incorrect identification of two streams at *r* = 0 and *r* = 2 mm in Case 1 and Case 3, the residual correction errors were only −0.27% and −0.89%.

The above three cases studies show that the measurement error is highly profile-dependent, and this is one of the reasons that such sensors can perform well only in relatively stable flow conditions once the sensors are calibrated in situ.

## 7. Effect of Velocity Profile

It is clear from Equation (2) that velocity profile will affect the accuracy of the proposed tail method. In order to evaluate the degree of effect, analysis was conducted under the following conditions: all flow streams passing through the pipe cross-sectional area carry the same power, and the velocity of each stream follows a turbulent velocity profile determined by the following equation:

(16) Vr=VC(1−rR)1n

where *V_r_* is the velocity at radial path *r*, *V_C_* is the velocity at the pipe center where *r* = *0*. *R* is the radius of the pipe and *n* is used to indicate the level of turbulence. A larger *n* indicates a flatter velocity distribution.

**Figure 7:**
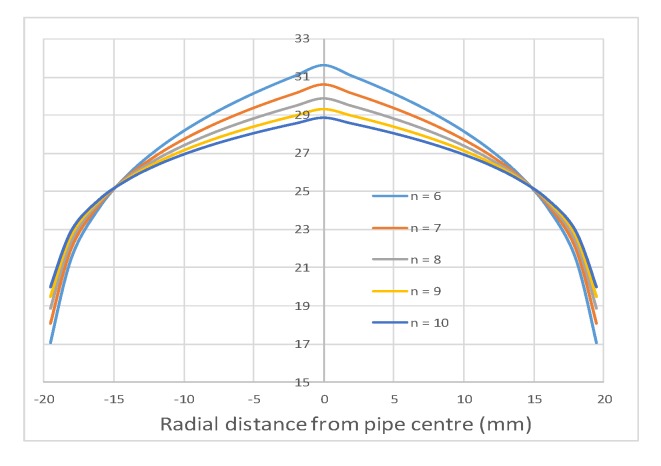
Turbulent flow profiles.

The method proposed here was developed under the condition of a turbulent velocity profile for *n* = 10 with the mean velocity 25 ms^−1^. Under the same mean velocity of 25 ms^−1^ and with variation of the turbulence *n* from 6 to 10, the errors were calculated based on the interpolations developed under the velocity profile for *n* = 10. The graphs in Figure 7 show the velocity profiles under different turbulence levels for the same mean velocity of 25 ms^−1^.

Table 5 provides the velocity profile data where *R*, the inner radius of the sensor, is 20 mm. It can be seen from Figure 7 and Table 6 that the higher the number *n,* the more uniform the velocity profiles across the cross-sectional areas.

## 8. Analysis Methodology

In the case presented here, the sensor was calibrated under the turbulent velocity profile for *n* = 10. Under different turbulence level, for each successive tail of the overall power spectrum, the streams radial paths were incorrectly identified using factor *β*, and in turn, radial path deification error occurred.

The following case describes a simple scenario where at each chosen radial path, a follow stream of 1 unit passes through the sensor, and the velocity at a given radial path is determined by the turbulence level under discussion, which varies from 6 to 10.

Compared to the actual total input power contained, the error can be estimated if interpolation is based on the velocity profile for *n* = 10. Table 7 provides the results.

Table 7 shows that due to effect of velocity profile change, a input flow stream with 1 unit power for *n* = 10 at *r* = 0 mm could be incorrectly recognized as 0.934 units for *n* = 9, 1.084 units for *n* = 8, 0.844 units for *n* = 7, and 0.787 units for *n* = 6. In terms of the total equivalent power, the error ranged from about 2% to about 8% with a mild velocity profile change from *n* = 10 to *n* = 9, to a significant velocity profile change from *n* = 10 to *n* = 6.

## 9. Discussions

From the above analysis, it is clear that the simulation results were in agreement with the experimental results for the charge induction on the electrode due to a single charged particle based on which the mathematical model was developed. The spatial sensitivity with regard to the flow stream was derived. The tail method was proposed and the methodology was demonstrated though examples. It was apparent that the successive tails of the overall power density functions of the sensor’s output were dominated by the outmost layer responses. The advantage of the tail method is that with a simple and robust ring-shaped sensor structure, the uniformity of spatial sensitivity can be greatly improved through digital signal processing; this is a low cost approach compared to tomographic systems.

To achieve the required accuracy, the number of points in the tail and the radial resolution should be adapted according to radial paths, sampling length, and fitting accuracy requirement. Residual error after the compensation depends on the degree of difference between the actual and the calibration velocity profiles. As the analysis results indicated, when the velocity profile varies, a good accuracy can still be achieved if the flow turbulence level varies from *n* = 8 to *n* = 10. If the velocity profile has also been obtained, the tail method can be more effectively adopted for industrial applications. For example, two arrays of electrostatic electrodes can be used upstream and downstream to measure velocity profiles, and these arrays can be electronically connected to form a ring at the same time, so that the techniques proposed here can be embedded in a time-sharing system. Data fusion or sensor modality should be pursued in future developments.

## 10. Conclusions

In summary, a novel signal processing method to improve the uniformity of the spatial sensitivity was proposed, and the simulations showed very promising results. The non-uniform spatial sensitivity not only caused measurement errors, but also prevented sensors from being applied under various flow conditions. With further development of this technique via data fusion and modality sensors, a substantial improvement in gas–solid flow measurements using ring-shaped sensors can be expected.

The method presented here is for a ring-shaped electrostatic sensor; however, the concept could also be applied in other sensing systems.

## Figures and Tables

**Table 1:** Parameters obtained from modeling.

Radial Position *r* (mm)	*α*(*r*) (Normalized)	*β*(*r*) *s*^2^ rad^−2^
0	1	2.32 × 10^−7^
2	0.9948	2.28 × 10^−7^
4	0.9803	2.18 × 10^−7^
6	0.9768	1.99 × 10^−7^
8	0.9616	1.71 × 10^−7^
10	0.9413	1.40 × 10^−7^
12	0.8793	9.96 × 10^−8^
14	0.8519	6.57 × 10^−8^
16	0.8185	3.42 × 10^−8^
18	0.8706	1.31 × 10^−8^

**Table 2:** Parameters obtained from modeling.

Radial Position *r* (mm)	Correction Factor in Terms of Total Power
0	64.94
2	63.58
4	60.44
6	52.71
8	42.93
10	32.20
12	20.70
14	11.41
16	4.48
18	1

**Table 3:** Decomposition.

Radial Path *r* (mm)	*μ* (Normalized)	*β s^2^/rad^2^*
0	1	2.32 × 10^−7^
10	4.707	1.40 × 10^−7^
14	8.519	6.57 × 10^−8^
16	4.911	3.42 × 10^−8^

**Table 4:** Interpolation.

***β* Range**	**Interpolation *r***
1.31 × 10^−8^–9.96 × 10^−8^	*r* = −5.9217 × (10^7^*β*)^3^ + 12.577 × (10^7^*β*)^2^ − 14.367 × 10^7^*β* + 19.682
9.96 × 10^−8^–2.19 × 10^−7^	*r* = −4.6065 × (10^7^*β*)^4^ + 28.083 × (10^7^*β*)^3^ − 64.739 × (10^7^*β*)^2^ + 60.676 × 10^7^*β* − 7.4253
2.19 × 10^−7^–2.28 × 10^−7^	*r* = −20.529 × 10^7^*β* + 48.897
2.28 × 10^−7^–2.32 × 10^−7^	*r* = 114.819−49.3868 × 10^7^*β*
Interpolation of *α*	
***r* Range**	**Interpolation *α***
0–10 mm	*α* = −0.0057*r* + 1.0041
10–14 mm	*α* = 0.0043*r*^2^ − 0.1265*r* + 1.7727
14–18 mm	*α* = 0.0107*r*^2^ − 0.3376*r* + 3.4823

**Table 5:** Error estimation.

Radial Paths r (mm)	0	2	4	6	8	10	12	14	16	18	Total Power	Error (%)
Correction factor	64.94	63.58	60.44	52.71	42.93	32.20	20.70	11.41	4.48	1		
Case 1												
Input stream intensity	1	1	1	1	1	1	1	1	1	1		
Original power	1.032	1.054	1.109	1.272	1.561	2.081	3.238	5.872	14.953	67.03	99.20	−85.2
Theoretically corrected Power	67.03	67.03	67.03	67.03	67.03	67.03	67.03	67.03	67.03	67.03	**670.30**	
Actually corrected power		132.26 *	67.03	67.03	67.03	67.03	67.03	67.03	67.03	67.03	668.50	−0.27
Case 2												
Input stream intensity	1	0	0	0	0	5	0	10	6	0		
Original power	1.032	0	0	0	0	10.41	0	58.72	89.72	0	159.88	−89.2
Theoretically corrected power	67.03	0	0	0	0	335.16	0	670.31	402.19		1474.69	
Actually corrected power	67.03	0	0	0	0	335.16	0	670.31	402.19		1474.69	0.00
Case 3												
Input stream intensity	1	1								1		
Original power	1.032	1.054								67.03	69.11	−65.6
Theoretically corrected power	67.03	67.03	0	0	0	0	0	0	0	67.03	201.09	
Actually corrected power		132.26 *	0	0	0	0	0	0	0	67.03		−0.89

* the flow streams at *r* = 0 and *r* = 1 mm were combined into one stream at *r* = 0.9978 mm.

**Table 6:** Velocity profile data.

V_mean_ = 25 m/s					
Turbulent Level	n = 6	n = 7	n = 8	n = 9	n = 10
r/R	*Vr*	*Vr*	*Vr*	*Vr*	*Vr*
0	31.60	30.61	29.88	29.32	28.88
0.1	31.05	30.15	29.49	28.98	28.57
0.2	30.44	29.65	29.06	28.60	28.24
0.3	29.77	29.09	28.58	28.18	27.86
0.4	29.02	28.46	28.03	27.70	27.44
0.5	28.15	27.73	27.40	27.15	26.94
0.6	27.12	26.86	26.65	26.48	26.35
0.7	25.85	25.77	25.71	25.65	25.60
0.8	24.16	24.32	24.44	24.52	24.58
0.9	21.53	22.03	22.41	22.70	22.94
0.975	17.09	18.07	18.84	19.46	19.97

**Table 7:** Effect of velocity profile.

Radial Path	n = 10	n = 9	n = 8	n = 7	n = 6
r	input power	eq. input power	eq. input power	eq. input power	eq. input power
0	1	0.934	1.084	0.844	0.787
2	1	0.956	1.053	0.867	0.813
4	1	0.957	1.080	0.883	0.832
6	1	0.975	1.084	0.908	0.856
8	1	0.973	1.041	0.916	0.879
10	1	0.983	1.019	0.944	0.912
12	1	0.989	1.008	0.960	0.940
14	1	0.997	1.001	0.987	0.980
16	1	1.006	0.998	1.022	1.037
18	1	1.028	0.996	1.107	1.172
Total input	10	9.799	10.366	9.439	9.209
Error %	0	−2.014	3.660	−5.611	−7.910
